# Blockchain-Based Smart Home Networks Security Empowered with Fused Machine Learning

**DOI:** 10.3390/s22124522

**Published:** 2022-06-15

**Authors:** Muhammad Sajid Farooq, Safiullah Khan, Abdur Rehman, Sagheer Abbas, Muhammad Adnan Khan, Seong Oun Hwang

**Affiliations:** 1 School of Computer Science, National College of Business Administration & Economics, Lahore 54000, Pakistan; msajidfarooq@lgu.edu.pk (M.S.F.); arbhatti@ncbae.edu.pk (A.R.); dr.sagheer@ncbae.edu.pk (S.A.); 2 Department of IT Convergence Engineering, Gachon University, Seongnam 13120, Korea; safi@gachon.ac.kr; 3 Pattern Recognition and Machine Learning Lab, Department of Software, Gachon University, Seongnam 13557, Korea; 4 Department of Computer Engineering, Gachon University, Seongnam 13120, Korea

**Keywords:** Real-Time Sequential Deep Extreme Learning Machine, data fusion, blockchain, smart home

## Abstract

Security and privacy in the Internet of Things (IoT) other significant challenges, primarily because of the vast scale and deployment of IoT networks. Blockchain-based solutions support decentralized protection and privacy. In this study, a private blockchain-based smart home network architecture for estimating intrusion detection empowered with a Fused Real-Time Sequential Deep Extreme Learning Machine (RTS-DELM) system model is proposed. This study investigates the methodology of RTS-DELM implemented in blockchain-based smart homes to detect any malicious activity. The approach of data fusion and the decision level fusion technique are also implemented to achieve enhanced accuracy. This study examines the numerous key components and features of the smart home network framework more extensively. The Fused RTS-DELM technique achieves a very significant level of stability with a low error rate for any intrusion activity in smart home networks. The simulation findings indicate that this suggested technique successfully optimizes smart home networks for monitoring and detecting harmful or intrusive activities.

## 1. Introduction

A smart home is connected to the Internet, allowing users to manage a variety of smart gadgets, each of which serves an important purpose in the home for the user and their family. The IoT is the foundation of an intelligent home network, connecting disparate intelligent devices such as smartphones, smart computers, and wearable devices. Citizens’ lives can be made easier and safer by making their homes more open and secure. The smart home provides useful resources such as monitoring habits and even safety tests, which have compelled consumers and system developers to conduct extensive research.

Blockchain-type systems and unified “cloud-like” computing networks can be used to solve these problems. Blockchain was developed in 2008 by Satoshi Nakamoto and includes a time-stamped set of malicious evidence documentation managed by a network of autonomous networks [1]. Blockchain architecture consists of a series of blocks linked together by simple cryptography. The three main concepts underlying the operation of blockchain technologies are inflexibility, decentralization, and transparency. The three roles have been remarkably effective, exposing them to a wide range of digital currency technologies, such as the functionality of mobile vehicles, mobile phones, and embedded systems. While the blockchain platform is secure and anonymous, there are some issues with its current implementation. For example, Sybil attacks by generations of false identities to manipulate the community have become more complex.

Since standard methods only look at the signatures and do not operate on searching for various specific patterns, a robust intrusion detection system is essential to analyze the circumstances thoroughly. RTS-DELM is a machine learning technique used to analyze data. This machine learning program uses an automated dataflow framework to determine data flow to detect intrusions and attack patterns. To handle the continually emerging smart blockchain-based applications, it is important to create powerful and versatile algorithms. Machine learning is a method that includes computers that teach themselves using an intelligent algorithm. According to one argument, machine learning is one of the first use cases of Artificial Intelligence (AI). The theory of machine learning helps machines to solve tasks without being explicitly programmed. The major objective of this sort of study is to develop a realistic algorithm that will receive information from the input and forecast it, as well as altering the outputs using statistical analysis. By utilizing machine learning, one can process a massive amount of data and arrive at a judgment based on facts.

Furthermore, we suggest that a smart home network architecture that will overcome the current problems related to the centralized security of home networks and will address future attacks on smart home networks. In the present study, an RTS-DELM methodology is used to make smarter homes safer using Internet of Things (IoT) powered sensors with enhanced efficiency. The key contributions to this research include a comprehensive overview of technological innovations applicable to blockchain-based smart homes empowered by RTS-DELM and a new outlook on diverse implementations (e.g., smart home data sharing), assisted by the recent stages of technological advancement.

RTS-DELM enables the automation of data analytics procedures and the generation of real-time insights. The datasets that the RTS-DELM methodology can assess can be manipulated in smart home networks, which means that all inaccuracies can be eliminated. Networks require consistent data. Any data-related issues in the RTS-DELM system will be ignored. It contains a method for detecting and anticipating possible deception and other unlawful activities. The purpose of this article is to examine an RTS-DELM-based system model for the smart estimation of intrusion detection in smart home networks with the highest degree of reliability. In the training and testing of intrusion detection in smart home network optimization with RTS-DELM, the fused datasets NSL-KDD (123,323 data samples) and KDD CUP 99 (25,193 data samples) are analyzed so that every instance has specific and varied features. As a result, the analysis and comparison of the finest approaches take place in the same place.

Due to the enormous amount of information transmitted in complicated networks, data fusion algorithms can be of benefit. A data fusion operation can transform many communications into useful and accurate data for the end-user. This article offers a data fusion technique for networks that grow organically to huge numbers of nodes. Numerous strategies and concepts for data fusion have been discussed in recent academic literature. “Data fusion” and “information fusion” are two of the most often used categories. Our investigation is limited to data supplied by sensors and not to data collected by any other source. Sensors are employed in a data fusion method to improve the accuracy of outcomes. The suggested approach secures the blockchain-based smart home system by carefully evaluating its dependability concerning the important security objectives of secrecy, authenticity, and usability. Furthermore, we assess our method’s ability to efficiently secure sensitive information while using relatively little power to show our argument that the overhead generated by our method is negligibly connected to the value of its security and privacy.

The remainder of this article consists of the following parts. Section 2 contains survey papers of related studies. Section 3 explains the underlying blockchain technologies and introduces an RTS-DELM solution for blockchain-centered mobile home and smart home application systems. Section 4 presents an approach to the RTS-DELM problem, including the simulation and findings of the DELM method. Finally, Section 5 addresses the conclusions of the study found from the details.

## 2. Literature Review

Blockchain is a current buzzword among smart home enthusiasts, and a range of research articles have been produced on the subject. In research exploring how blockchain technologies would be used in the smart city, S. Aggarwal et al. [2] discussed many aspects of healthcare, including transaction assimilation, home healthcare, and investment sharing. Nonetheless, the application of blockchain technologies in the smart home has not been explored comprehensively in information analysis. In many ways, the blockchain can be used in the smart home sector. M. Andoni et al. [3] provided a detailed study of different blockchain applications of a peer-to-peer resource sharing network. The study presents detailed information on the implementation and capabilities of various smart home networks, such as security challenges in the smart grid, big data analysis, Artificial Intelligence (AI), and payment systems. However, their study did not adequately account for smart house-related issues, such as smart home security and smart city financial planning.

Khan et al. [4] suggested a user-based blockchain structure to secure the connectivity of edge information in the Internet of Things. Z. Zhou et al. [5] researched blockchain technologies, contractual analysis, and distributed computing to transfer the control and performance of certain automobiles. J. Wu et al. [6] suggested a software-specified blockchain interface to recognize dynamic blockchain frameworks and proceeded to apply a consent function approach to virtual machines with an application-aware system that can extract and manage unique consensus resources.

Sivaraman et al. [7] examined security issues in the smart home networks and made constructive suggestions. It is necessary to monitor and validate the systems that have been approved with an algorithm server and run the smart home equipment also in the external internet world. Via the abolition of a requirement for recognizing users and banning data packets that are not originating from the internet, the current regulations cannot efficiently protect internal user data. Lee et al. [8] introduced an upgrade that handles software upgrades of embedded systems utilizing a blockchain, cryptographic certificates that are implemented with shared keys, and introduces encryption protocols utilizing a private key. A smart house has several tiny, embedded devices that are linked to each other. However, to utilize this mechanism, there are more components required than what is needed for a home.

Nevertheless, this method is less effective in the absence of adequate learning data to generate a machine learning prototype. Panwar et al. [9] researched different cybersecurity risks in the smart home and their responses. They offer a detailed description of smart home defense in different forms of threats. Khan et al. [10] contributed a confidentiality and integrity strategy. This sensor offers safe data collection, encryption, and inquiries for smart home applications. It preserves the information exchanged between the individual, gateway, network operator, and the system, thus promoting information verification and privacy. Hsu et al. [11] proposed a multisensory data fusion technique within the smart home methodology. They developed an intelligent smart home environment using smart wearable devices to control and operate the services of the smart home.

In the current digital era, the exponential growth of Internet of Things (IoT) devices raises various security and privacy-related design challenges for businesses. Previous research indicates that blockchain technology is a key answer to the IoT’s data security problems. The blockchain method allows multiple data providers to share information safely and reliably. IoT data are encrypted and stored in a distributed ledger [12]. Wang et al. [13] investigated the security threats connected with data storage in sensor networks before proposing the use of blockchain technology to guarantee the security of data stored in sensor networks. The work provides both member proof and non-member proof by employing a cryptographic accumulator instead of a Merkle hash tree. In addition, the number of items in the current accumulator is restricted and cannot match the growing requirements of the blockchain. Therefore, the authors suggest a new form of the unbounded accumulator, together with its description and security model. Finally, using bilinear pairings, an unbounded accumulator system is constructed, and its performance is evaluated.

Bordel et al. [14] offer a theoretical framework for trust in IoT situations, including a formalization in mathematics and a description of the conditions that a solution for trust provision must satisfy. A study of these requirements reveals that blockchain technology fully satisfies them; thus, a first trust provision system based on blockchain networks is also presented. To evaluate the approach given, experimental validation is also proposed and carried out. Ra et al. [15] suggested a method for password-protected secret sharing key retrieval, secured by a secure password from a malicious key storage server of a PBC. Existing systems for key recovery and password-protected secret exchange are described.

Singh et al. [16] proposed DeepBlockScheme: a deep learning-based blockchain-driven scheme for a secure smart city, in which the blockchain is deployed at the fog layer to assure manufacturing data integrity, decentralization, and security. Deep learning is used at the cloud layer to boost productivity, automate data processing, and boost communication bandwidth in smart factory and smart manufacturing applications. Zhang et al. [17] presented a strategy that uses an expiry recognition approach based on the Least Recently Used (LRU) algorithm to divide the blockchain transaction database into cold and hot zones. It can save space by transferring unspent transaction outputs outside of in-memory transaction databases. Salim et al. [18] proposed a quick, efficient handover authentication technique that uses deep learning to authenticate devices and construct a user profile-based system for instant permission. The channel state data of a user’s movement pattern train the model and identify bad users impersonating honest users.

## 3. Proposed Methodology

A. Background

In the year 2008, Satoshi Nakamoto invented the blockchain. A blockchain-based peer-to-peer payments system can eradicate intermediaries and double-spends by using a primary cryptocurrency (e.g., bitcoins). It is a centralized method where any information block is validated by SHA-256 (Secure Hash Algorithm) using a former hash block. The block contains a large amount of transaction stored data such as the block number of the previous block hash, transaction data, a nonce, and time stamps. The timestamp contains a constant parameter, but a nonce parameter is randomly generated. The miner (computer module) has control over the static (block) and dynamic (timestamp and nonce) portions of an information chain and calculates the leading number of zeros required to form the header of the block.

**Figure 1:**
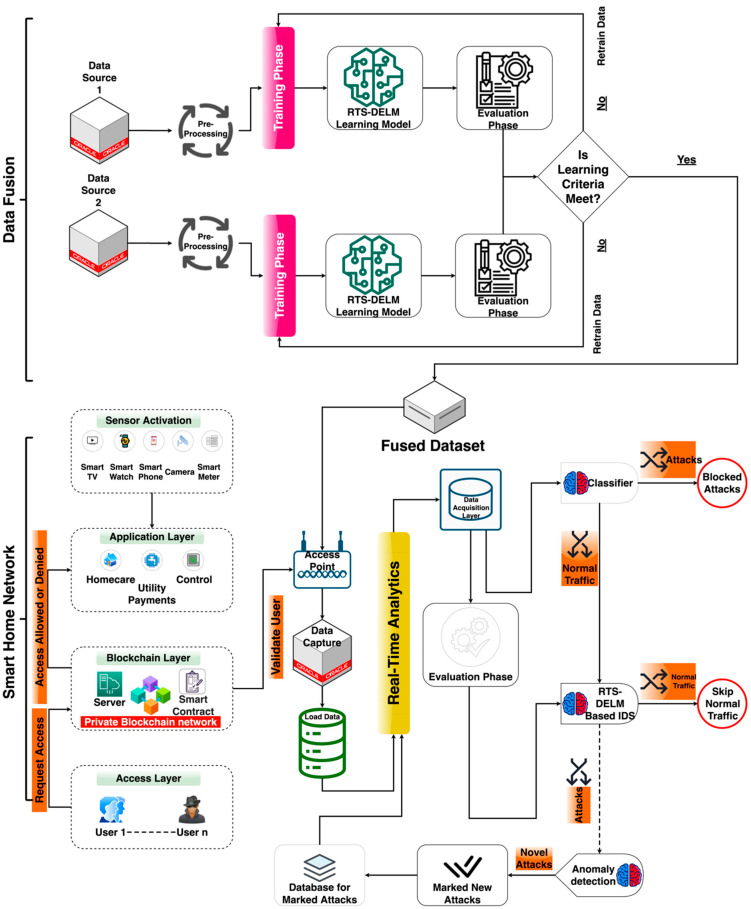
A proposed blockchain-based smart home network.

Figure 1 highlights the smart home network centered on the private blockchain network. The platform includes four layers: a layer of data sources connecting to the network and emitting user information, a private blockchain network layer empowered with RTS-DELM that unleashes predictive analysis against thousands of files, a client node with smart home system data, and a collection of home devices to simulate the data.

B. Integration Data Fusion Technique in Blockchain-Based Smart Home

Data fusion approaches incorporate information from various sensors to obtain more accurate observations than could be accomplished by using a single, separate sensor. Information extraction is the practice of extracting information from different and probably interconnected sources and integrating it in a way that will get the most impactful outcomes. For example, a series of network security sensors are in operation in a security framework. It is impossible to achieve a wide-angle, all-encompassing image of the overall security state of a dynamic system of the security system. Furthermore, devices that are spread over an extensive range can be challenging to manage effectively. To increase the efficiency of the model and provide analysis with an entire system protection condition, it is also essential to effectively and intelligently fuse the outcomes of these sensors.

In comparison, several data sources can offer more consistent reliability because the information itself comes from various sources. Consequently, data fusion approaches by integrating information from various data sources can produce more reliable and stable results than those obtained by a single information source. In this manner, the NSL-KDD [19] and KDD CUP 99 [20] datasets were used to evaluate the suggested system’s performance—these datasets were utilized to perform data fusion. Each data collection specifies a unique link that corresponds to a sequence of packets that flow between the provider and target locations in the fused data collection according to a predefined protocol. This data collection has 41 characteristics per record. Six distinct fields and 35 continuous fields compose these features.

C. Integration of Real-Time Sequential Deep Extreme Learning Machine in Blockchain-based Smart Home

Listed below are the advantages when RTS-DELM is implemented;

- User authentication, as a means to legitimately access and make transactions on the blockchain network.
- Blockchain offers a high degree of trust and protection. Blockchain applications incorporate real-time transaction mechanisms into smart contracts to ensure that the contractual commitments, which were already negotiated, are fulfilled.
- Blockchain is a reliable way to incorporate an incentive-based mechanism to enable consumers and users to make a data contribution. In addition, this big data would help to refine the RTS-DELM model.

The usage of blockchain-based systems can be rendered smarter by the use of RTS-DELM computational technology. The confidentiality of data can be enhanced when utilizing the RTS-DELM distributed blockchain technology. RTS-DELM can also be used to increase the pace at which comprehension is achieved by exchanging further knowledge, thereby improving understanding. It offers the framework and network structure to develop a decentralized blockchain application. In this article, we analyze the RTS-DELM deployment architecture, which is an advanced system. The proper use of this technology will be to collect intelligence from different information resources, such as sensors, mobile devices, and IoT systems. Knowledge derived by utilizing these techniques is used for smart apps. The blockchain is the central structural feature of smart apps. Nonetheless, for analysis, the RTS-DELM method may be used to evaluate and forecast real-time data. The blockchain also processes all the data that might be required from the RTS-DELM model.

Data errors such as duplication, missing data parameters, glitches, and noise are reduced when making data for research. Knowledge is transmitted through the blockchain, and the minimization of data-related issues can be solved in the RTS-DELM framework. The RTS-DELM technique can function well where only a limited portion of a data set is needed. The architecture provides a wide variety of implementations in various areas, such as fraud detection and prevention. The blockchain infrastructure focuses on the edge of the Internet of Things (IoT) and comprises three main elements: blockchain layer, knowledge architecture, smart contracts, and the RTS-DELM framework. In the proposed RTS-DELM system, vast quantities of hidden layers, hidden neurons, and several activating mechanisms have been employed to optimize the privacy and protection of smart homes. There are three separate steps in analyzing the data in the proposed method: the data acquisition, preprocessing, and assessment stages. The evaluation layer was made up of two sub-layers: the prediction and performance layer. For analysis, accurate data are obtained from sensors and actuators. Then, the data are given as raw data and used by the collection layer. A comprehensive technique for data cleaning and preparing has been implemented to remove discrepancies in the preprocessing layer. The RTS-DELM was employed to maximize the home network protection by preventing disruptive or invasive applications.

Cryptographic hash functions connect blocks via cryptography. A home server computer could be viewed as a miner to validate new transactions and introduce new blocks. In contrast, intelligent contracts follow predefined rules to make decentralized transactions easier and quicker. There are diverse consensus models for blockchains, such as proprietary, public, and federated, but private blockchains are more effective when used in smart homes.

The deployment layer can provide interoperability between smart home devices and blockchain networks. The paper tackles wireless home technology, interoperability in the home, connectivity control, electronic billing of networks and municipal resources, and healthcare. Consequently, the access layer serves to interact with devices at the intersection layers such as at the individual level, the customer level (mainly for tech companies, including for marketing and use in retail), as well as for the corporate level as the topmost “layer” of the smart home, which primarily influences entities to take opportunity of the blockchain smart home ecosystem, which is a huge market.

With smart appliances, smart technology, and smart upgrades, smart home architecture is the tip of the iceberg; it is going to be a cornerstone of the smart home for smart households. Living in a smart home environment includes having a smart scan of one’s home with rooms, locking doors, smart devices that trigger conditions, and more. Through the functions provided with the smart home, the user can regulate remote electricity, set off alarms, watch and secure their home with video monitoring, and control their vehicle, among several more applications. A user-friendly, custom-integrated framework that would enable the homeowner to enable it on request should be introduced to increase the seamless activity of smart homes and detect any disruptive behaviors by hackers. For an IoT system, the user’s permission authorization can be listed in a collection of IoT system control records, a specified set of IoT system owner records, or a specified set of IoT system owner list records. Many sources that preserve trustworthy information must store those pieces of information to withstand the assaults of hostile attackers.

To explain how blockchains will contribute to secure access, we present the following explanation.

**Figure 2:**
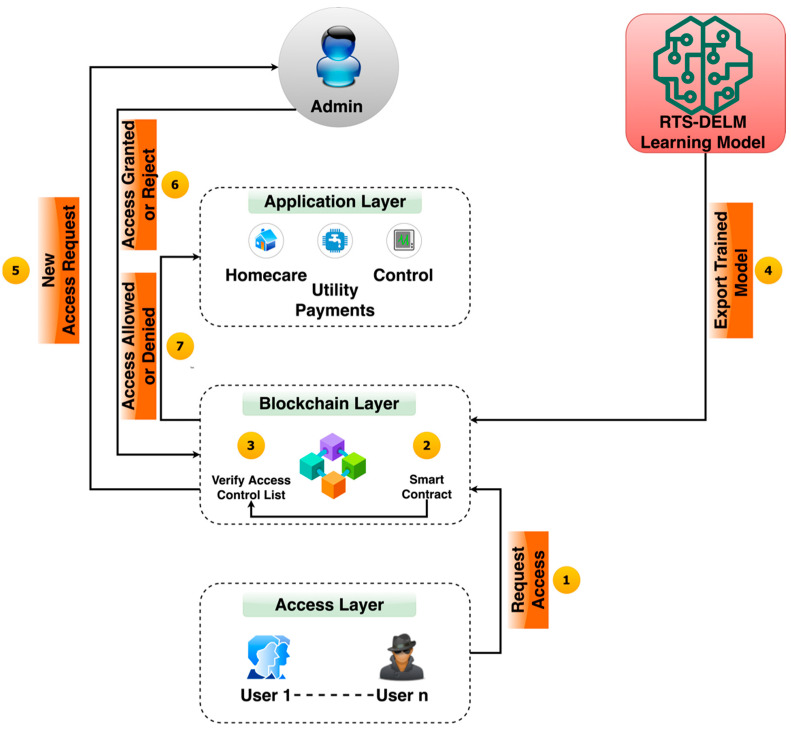
Proposed blockchain-based smart home management system.

- First, the consumer must decide the access level and add it to the home service computer. For example, at the highest level, the homeowner (Admin) is allowed, while teenagers, youths, visiting relatives, and adolescents need mid-level permission.
- For a user who has access to the smart home and is using applications inside, Figure 2 shows how blockchain facilitates secure entry.
- Relatives and visitors have relatively poor access permits. When processing a request from the client, the home server checks security access to repositories. Upon receiving an order from the customer, the home server transmits the encrypted username and password to the blockchain layer.
- For various users and implementations, a blockchain regulation header contains a set of authorization rules. The part of the block data used for applying control policies and services is the policy header.

The administrator checks the new user’s request and then accepts or rejects the access request. When the information is incorporated into the blockchain, miners take action depending on the policy specifics added to the header. This mechanism is successful in combatting malicious attackers.

D. Real-Time Sequential Deep Extreme Learning Machine

RTS-DELM combines a variety of hidden layers, multiple hidden neurons, and a variety of different activation functions to provide the ideal solution for improving smart home networks. The proposed technique comprises three different phases: gathering the data, reviewing it, and presenting it. We have two sub-layers in the application layer (there are also additional layers in-between), where one is for estimation and the other layer is for assessment. While experimenting, data were collected from sensors for observational investigation. The data obtained through the data collecting mechanism were then made available as input to the data collection mechanism. Until final processing, many data processing techniques were used to eliminate anomalies from the results. Finally, the RTS-DELM algorithm was utilized to improve smart home networks to prevent disruptive or intrusive behavior.

The RTS-DELM method applies to a wide variety of smart home applications. To maintain the requisite detection accuracy, a significant fraction of sensor readings is frequently required. RTS-DELM addresses a variety of network access concerns through the use of integrated routing and security measures. However, given that 80% of a network’s energy is used during data transmission and reception, data reduction and function abstraction approaches may help to reduce processing time and increase the endurance of neural networks. However, excessive compression will increase energy expenditures. Within smart home networks, RTS-DELM enables more efficient data compression. As a result, intelligent home networks require real-time networking solutions for security, scheduling, monitoring, clustering nodes, data aggregation, and fault diagnostics. The RTS-DELM design enables smart home networks to react smoothly to their changing environments.

In real-time, an RTS-DELM algorithm based on Deep Extreme Learning Machine (DELM) examines the data sequence. The DELM may be used in a variety of applications and domains to forecast health problems, assess energy consumption, inventory services, and specify transportation activities. The RTS-DELM may be used to categorize and regressively dedicate data in a variety of ways since it is intuitive and efficient at keeping up with the intricacy of frameworks. An extreme learning computer is a feed-forward neural network architecture that ensures that feedback can only shift one direction through those various layers, but “back-propagation” through the initial neural network can be consistently modified without error in the initial training process, where information passes through the initial neural network and advances in reverse through the corresponding error rate of achieving high precision at low errors. The weights in the model are constant throughout the validation phase of the system, in which the validated model is imported, the real data are predicted, and the precision of the model improves. There are several hidden layers and at least one output layer in the RTS-DELM model, as well as an input layer. Following conditioning, the framework is transported to the cloud for online use, and it is then utilized for validation services throughout the cloud during the validation process, as shown in the following diagram. In the assessment layer, the Mean Square Error (MSE) was examined to enhance smart home networks and other connected devices.

## 4. Simulation Results

The Fused RTS-DELM method was applied to the fused dataset in this research. The findings were distributed randomly between the training collection (126,238 samples) and the test/validation set (15% of the tests) (22,278 records). The data were evaluated in advance of their intended usage to ensure that they were error-free. The RTS-DELM method needed to know if its machines had been affected by ransomware or other cyber threats. Following that, we examined several neurons, including the activation of buried layers and numerous types of active activities. In research designed to determine performance, we successfully evaluated the production of RTS-DELM. To assess the RTS-DELM algorithm’s performance, we used a variety of statistical measures that explained the result.

(1) Misrate =(O1V0+O0V1)V0+V1

(2) Accuracy =(O0V0+O1V1)V0+V1

(3) Sepecificity =O0V0(O0V0+O0V1)

(4) Sensitivity =O1V1(O1V0+O1V1)

(5) $$\mathrm{True}\;\mathrm{Positive}\;\mathrm{Rate}\;\left(\mathrm{TPR}\right)=\frac{TP}{\left(TP+FN\right)}$$

(6) $$\mathrm{True}\;\mathrm{Negative}\;\mathrm{Rate}\;\left(\mathrm{TNR}\right)=\frac{TN}{\left(TN+FP\right)}$$

(7) $$\mathrm{Positive}\;\mathrm{Prediction}\;\mathrm{Value}\;\left(\mathrm{PPV}\right)=\frac{TP}{\left(TP+FP\right)}$$

(8) $$\mathrm{Negative}\;\mathrm{Prediction}\;\mathrm{Value}\;\left(\mathrm{NPV}\right)=\frac{TN}{\left(FN+TN\right)}$$

Table 1 shows the suggested RTS-DELM-based decentralized smart home network framework to predict intrusion detection during the training level. Throughout the training, a total of 126,238 recordings were used, divided into 65,495 and 60,743 normal and assault records, respectively. As a consequence, it is seen that the forecasting system properly anticipates 64,185 attack records of normal groups without a genuine attack while forecasting 1310 attack records incorrectly. In comparison, 60,743 records are obtained in the event of an attack, of which 58,707 are appropriately predicted as an attack and 2036 are incorrectly forecasted as a regular activity while the attack occurs.

Table 2 shows the suggested RTS-DELM-based decentralized smart home network framework to predict intrusion detection during validation level. Throughout the validation, a total of 22,278 records were used, divided into 11,558 normal and 10,720 assault records. It is seen that 11,072 records of normal class with no attack identified are properly predicted, whereas 486 records are forecasted falsely as having an attack, despite the absence of a genuine assault. In the event of a cyber-attack, 10,154 of the 10,720 data points collected were properly predicted as invasions, whereas 566 were incorrectly predicted as normal activities throughout the attack.

**Figure 3:**
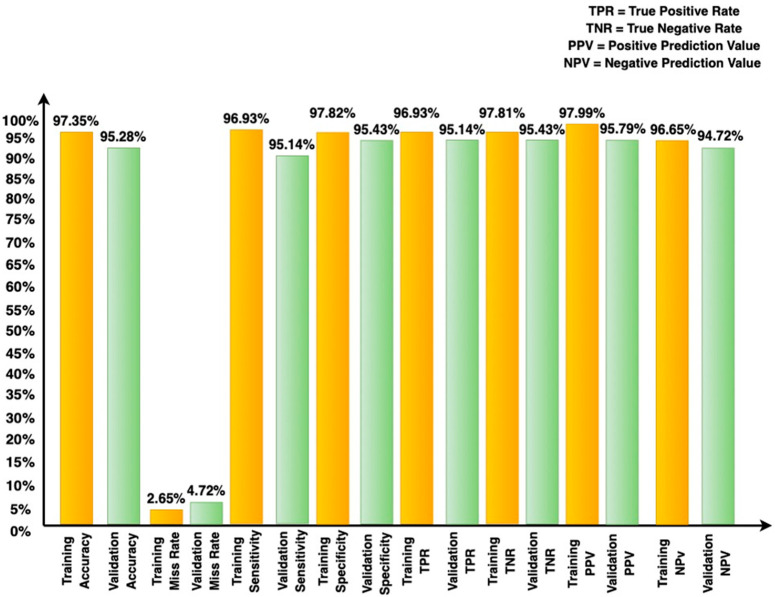
Different statistical measures for the proposed blockchain-based smart home network architecture for the estimation of intrusion with fused dataset during validation and training.

Figure 3 shows the assessment of the proposed RTS-DELM-based decentralized smart home network framework in terms of accuracy and the misclassification rate at the training and validation levels. It was proven that the proposed RTS-DELM-based decentralized smart home network system achieves a combined accuracy and misclassification rate of 97.35% and 2.65%, respectively, during training. Additionally, during validation, the proposed RTS-DELM decentralized smart home network system achieves a combined accuracy and misclassification rate of 95.28% and 4.72%, respectively.

Figure 3 illustrates the proposed blockchain-based smart home network security system’s effectiveness in various statistical parameters during the training and validation phases using the Fused RTS-DELM proposed framework. This demonstrates unequivocally that the suggested method generates accuracy and misclassification rates of 97.35% and 2.65%, respectively, during training. During validation, the suggested system achieves an accuracy rate of 95.28% and a misclassification rate of 4.72%. Additionally, it demonstrates the performance of the system model in terms of sensitivity, specificity, and true positive rate. (TPR), true negative rate (TNR), positive predicted value (PPV), and negative predicted value (NPV) during the training and validation phases.

We evaluated the dependability of our technique on the reliability of other published algorithms in the literature; in addition, as shown in Table 3, the suggested framework achieves much higher accuracy by reducing the error rate. In terms of accuracy, the proposed RTS-DELM framework outperforms existing algorithms already in use, such as the Artificial Neural Network-based Intrusion Detection System [21] and Generative Adversarial Networks (GANs) [22] and Deep Extreme Learning Machine (DELM) [4]. When compared to the DELM technique, the suggested RTS-DELM method achieves higher efficiency on the fused dataset because of the enhanced accuracy achieved [4]. In [21], the authors suggested an Artificial Neural Network-based Intrusion Detection System, and, in this method, the researchers achieved 81.2% precision. In [22], the researchers suggested Generative Adversarial Networks (GANs), and in this method, the researchers achieved 86.5% accuracy. Finally, in the DELM approach without data fusion, 93.91% accuracy was achieved [4]. In this study, the RTS-DELM system achieved an accuracy of 95.28%, which is greater than prior attempts, demonstrating its efficacy and demonstrating that the system performance is improved by employing the data fusion technique. The suggested RTS-DELM paradigm provides a much higher return on investment than existing methods. As a result, the RTS-DELM paradigm that has been proposed gives a viable solution to the aforementioned issue.

## 5. Conclusions

In this study, the idea of a smart contract in blockchain technology is employed to validate the user’s identity for accessibility to centralized smart home services. The most significant benefit of this research is the demonstration of how easy it is to receive facilities and how secure the resources are. There is no need to have redundant authentication because no other third-party users can access smart home systems, even if another user tries to access an already used resource. Intrusion detection in smart homes, especially in the context of assessment and prediction, remains a key concern. In the meantime, recent advances in the blockchain and machine learning sectors have demonstrated tremendous promise to accomplish these aims. Discussing the need for an efficient approach, this study provided a compact and efficient mechanism for intrusion prevention. An RTS-DELM approach was developed, and also data fusion techniques were presented to optimize multi-sensor networks. Numerous measures were used to assess the feasibility of the proposal. The consistency of RTS-DELM findings showed that the proposed method is more successful than others. The suggested RTS-DELM solution obtained an exceptionally high rate of success, showing 95.28% accuracy. The findings obtained are encouraging, and we will continue to investigate more applications for the device through the deployment of more datasets and varying frameworks.

## Figures and Tables

**Table 1:** Training of the proposed blockchain-based smart home network architecture for the estimation of intrusion with fused dataset empowered with Fused RTS-DELM.

Suggested RTS-DELM-Based System Model (85% of Data)			
Total No. of Records (N = 126,238)		Outcome (Output)	
Input	Predictable outcome	(Normal) *O*_0_	(Attack) *O*_1_
	*V*_0_ = 65,495 Normal	64,185 (TP)	1310 (FP)
	*V*_1_ = 60,743 Attack	2036 (FN)	58,707 (TN)

**Table 2:** Validation of the proposed blockchain-based smart home network architecture for the estimation of intrusion with fused dataset empowered with Fused RTS-DELM.

Suggested RTS-DELM-Based System Model (15% of Data)			
Total No. of Records (N = 22,278)		Outcome (Output)	
Input	Predictable outcome (*V*_0_, *V*_1_)	(Normal) *O*_0_	(Attack) *O*_1_
	*V*_0_ = 11,558 Normal	11,072 (TP)	486 (FP)
	*V*_1_ = 10,720 Attack	566 (FN)	10,154 (TN)

**Table 3:** Comparison of results of the proposed data fusion technique of decentralized smart home network based on Fused RTS-DELM with the literature.

Method	Accuracy Rate
ANN Based IDS [21]	81.2%
GAN [22]	86.5%
DELM [4]	93.91%
Fused RTS-DELM with Data Fusion (Proposed Blockchain Model)	95.28%

## Data Availability

The simulation files/data used to support the findings of this study are available from the corresponding author upon request.
